# Dementia resulting from traumatic brain injury

**DOI:** 10.1590/1980-57642015DN94000356

**Published:** 2015

**Authors:** Joana Ramalho, Mauricio Castillo

**Affiliations:** 1 Centro Hospitalar de Lisboa Central, Lisboa, Portugal; University of North Carolina at Chapel Hill, Chapel Hill, NC, US.; 2 University of North Carolina at Chapel Hill, Chapel Hill, NC, US.

**Keywords:** craniocerebral trauma, chronic traumatic encephalopathy, post-concussion syndrome, dementia, magnetic resonance, traumatismo cranioencefálico, encefalopatia crônica pós-traumática, síndrome pós-concussão, demência, ressonância magnética

## Abstract

Traumatic brain injury (TBI) represents a significant public health problem in modern societies. It is primarily a consequence of traffic-related accidents and falls. Other recently recognized causes include sports injuries and indirect forces such as shock waves from battlefield explosions. TBI is an important cause of death and lifelong disability and represents the most well-established environmental risk factor for dementia. With the growing recognition that even mild head injury can lead to neurocognitive deficits, imaging of brain injury has assumed greater importance. However, there is no single imaging modality capable of characterizing TBI. Current advances, particularly in MR imaging, enable visualization and quantification of structural and functional brain changes not hitherto possible. In this review, we summarize data linking TBI with dementia, emphasizing the imaging techniques currently available in clinical practice along with some advances in medical knowledge.

## Abstract (pt)

O traumatismo cranioencefálico (TCE) representa um importante problema de saúde pública nas sociedades modernas. As suas principais causas são: os acidentes de trânsito e as quedas. O traumatismo leve e repetido relacionado com os esportes de contato ou o traumatismo relacionado com as ondas de choque provenientes de explosões em cenário de guerra são hoje reconhecidas como importantes causas de TCE. A mortalidade e morbilidade associada ao TCE é considerável. TCE representa o fator de risco ambiental melhor reconhecido para o desenvolvimento de demência. Com o reconhecimento recente de que até o TCE leve pode determinar déficts cognitivos, os estudos de imagem adquiriram grande importância neste contexto. Contudo, não está definido qual o melhor estudo de imagem para caracterizar o TCE. Avanços tecnológicos, como a ressonância magnética, permitem atualmente identificar e quantificar alterações intra-parenquimatosas estruturais e funcionais, não detectáveis nos estudos convencionais. Neste artigo os autores resumem os estudos que relacionam TCE e demência, dando particular ênfase às técnicas de imagem atualmente disponíveis na prática clínica, bem como alguns avanços nos métodos de imagem ainda limitados ao plano da investigação.

## INTRODUCTION

Traumatic brain injury (TBI) is a common clinical problem that represents a major source of morbidity and mortality in the modern era. The most common causes of head injury are motor vehicle accidents and falls. TBI is commonly classified as mild, moderate or severe, depending on whether the injury causes unconsciousness, how long unconsciousness lasts and the severity of symptoms. Although most TBIs are classified as mild because they are not life-threatening, even a mild TBI can have serious and long-lasting effects. One of the most feared long-term consequence of TBI is dementia, since a number of epidemiologic studies have shown that experiencing a TBI in early or midlife is associated with an increased risk of dementia in later life.^1^ Common areas of cognitive impairment caused by TBI include memory, information processing speed, attention and executive function, with many TBI patients experiencing degradation in all four even when their TBIs have been classified as mild.^2^ It has also been observed that repetitive mild head trauma, as experienced by professional boxers, football and hockey players, is associated with a high risk of chronic traumatic encephalopathy (CTE) a type of dementia with distinctive clinical and pathologic features. The recent recognition that a similar syndrome may occur in military personnel, who also experience high rates of mild TBI, has rekindled interest in this condition.^1^

Long-term cognitive deficits may occur despite normal-appearing brain on conventional neuroimaging studies, such as computed tomography (CT) and magnetic resonance (MR) imaging. In this setting, advanced MR techniques, including susceptibility-weighted imaging (SWI), diffusion-weighted imaging (DWI), perfusion-weighted imaging (PWI), magnetization transfer MRI (MT), magnetic resonance spectroscopy (MRS), diffusion tensor imaging (DTI) and functional blood oxygen level-dependent MRI (fMRI) may shed light on subtle but important findings in TBI. Other promising neuroimaging tools include positron emission tomography (PET), single photon emission tomography (SPECT) and magnetoencephalography (MEG). Our aim was to review data linking TBI with dementia and to summarize the brain-imaging techniques currently available in clinical practice along with some future imaging trends.

## DEFINITION/CLASSIFICATION

TBI occurs when an outside force hits the head hard enough to cause the brain to move violently within the skull disrupting normal brain function. According to whether the injury causes unconsciousness and how long unconsciousness lasts, brain injury may be classified as mild (mTBI) when no loss of consciousness occurs or unconsciousness lasts less than 30 minutes, moderate when unconsciousness lasts more than 30 minutes and severe if unconsciousness lasts more than 24 hours. TBI can also be classified using the Glasgow coma scale (GCS) as mild (GCS=13-15), moderate (GCS=9-12) and severe (GCS<8). However, defining minor versus major head injuries is complex. Some authors suggest major injury as worsening level of consciousness, loss of consciousness for more than 5 min, focal neurological findings, seizure, failure to improve in mental status over time, penetrating skull injuries, signs of a basal or depressed skull fracture, and confusion or aggression on examination.^3-8^

Post-concussion syndrome (PCS) refers to a set of nonspecific symptoms that may last for weeks, months, or years after a concussion. Its most common symptoms include headache, dizziness, difficulty concentrating, irritability, and sleep problems. Loss of consciousness is not required for a diagnosis of concussion or post-concussion syndrome. In most patients, post-concussion syndrome symptoms occur within the first seven to 10 days and resolve within three months but can persist for a year or longer.

Dementia is a loss of mental ability sufficiently severe to interfere with normal activities of daily living, lasting more than six months, not present at birth and not associated with a loss or alteration of consciousness. Indeed, dementia is not a single disease but an umbrella term covering a wide range of medical conditions that have in common a group of symptoms caused by gradual death of brain cells. The loss of cognitive abilities that occurs with dementia leads to impairments in memory, reasoning, planning and behavior. Brain changes that cause dementia may be temporary, but are more often permanent and worsen over time, leading to increasing disabilities and a shortened lifespan.^9^

Chronic traumatic encephalopathy (CTE) is a progressive degenerative disease associated with history of repetitive brain trauma including symptomatic concussions and asymptomatic subconcussive impacts to the head. This type of dementia was initially recognized in professional boxers by Harrison S. Martland in 1928,^10^ who described a group of signs and symptoms called *dementia pugilistica*. Currently CTE can only be definitively diagnosed postmortem and is classified as a distinct entity in the category of tauopathy. Tauopathies are characterized by dissociation of tau proteins from microtubules, which are hyperphosphorylated and aggregate to form neurofibrillary tangles (NFTs), a process that presumably serves as the instigator of toxicity and cell death by unknown mechanisms. CTE has been confirmed at autopsy in football, soccer, ice hockey, and other contact sports athletes who have an inherent risk of subconcussive or concussive injuries.^1,11,12^

## EPIDEMIOLOGY

An estimated 10 million people are affected annually by TBI worldwide.^13^ However, this figure is likely underestimated because it does not include patients with mTBI who frequently dismiss their symptoms and never seek medical attention (25%) or those who are seen in private clinics or by primary care physicians (14%) that do not report the clinical findings.^14,15^ The Centers for Disease Control and Prevention (CDC) estimate that 1.7 million Americans annually experience TBI severe enough to cause death or require emergency room care or hospitalization and that 5.3 million (almost 2% of the population) are living with some degree of TBI-related disabilities.^1^ According to the World Health Organization, TBI will surpass many diseases as the major cause of death and disability by the year 2020.^13,16^ Based on the large number of known and likely unknown cases, traumatic brain injury has been referred to as the "silent epidemic".^17^ Recently, the public has become more aware of TBI through news reports of sports injuries leading to long-term effects of repetitive trauma to the brain as well as news reports about soldiers returning from Iraq and Afghanistan with TBI which is now being called the "signature injury of war".^18,19^ These patients further increase the number of unaccounted cases of mild TBI since they do not seek medical attention in the belief that admission of symptoms may jeopardize their careers.^20,21^

It is estimated that 75-90% of all treated brain injuries are mild corresponding to an expected incidence of 100-300/100,000 cases according to the WHO task force.^22^ It has been proposed that as many as one in three mTBI victims have persistent long-term cognitive deficits,^23,24^ which can occur despite normal results on conventional neuroimaging studies. Based on currently available data, a rough calculation of how much of the population's burden of dementia is attributable to TBI suggests a risk of dementia attributable to TBI of 5%-15%.^1,25^

## ETIOLOGY

Leading causes of TBI in the general population include falls, motor vehicle collisions, assaults, and sports-related injuries.^21^ Falls are the leading cause of TBI in all ages. Those aged 75 years and older have the highest rates of TBI-related hospitalization, long-term cognitive changes, and death due to falls. Other recently recognized causes of TBI are repetitive head trauma resulting from contact sports, and combat-related head injuries seen in soldiers returning from war.^18^ In addition, TBI can result from bullet wounds or other injuries that penetrate the skull and brain.

**Figure 1:**
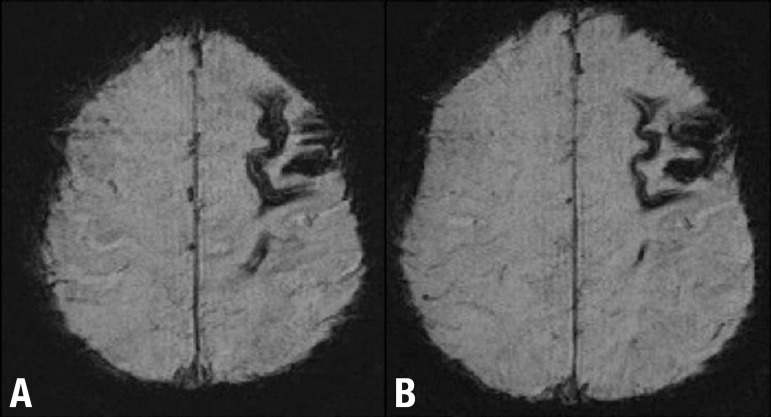
Patient with mild cognitive decline after moderate TBI. MR Susceptibility-weighted imaging (SWI) axial images [A and B] show frontal contusion and superficial siderosis seen as cortical low signal intensity lines over the cortex due to subarachnoid hemorrhage.

**Figure 2:**
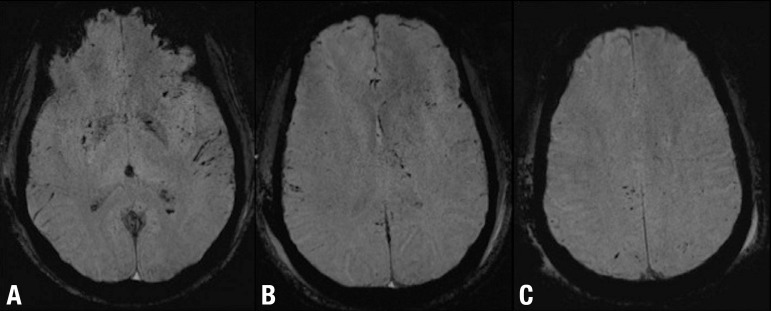
DAI and subarachnoid hemorrhage after mild TBI. Axial SWI [A, B and C] show multiple focal lesions involving the basal ganglia and the lobar white matter at the gray-white matter interface. Note also the bilateral subarachnoid hemorrhage particularly evident in A B C the left sylvian fissure.

**Figure 3:**
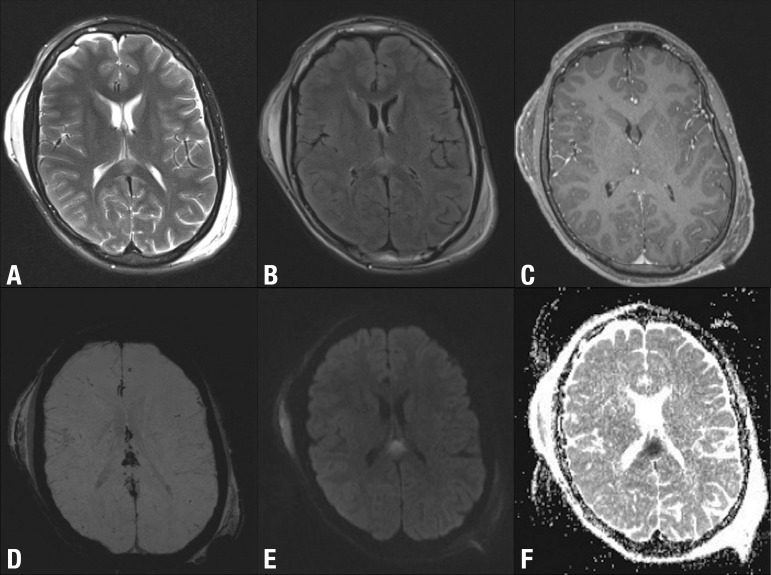
DAI involving the splenium of the corpus callosum after TBI. Axial T2-weighted [A], FLAIR [B], post-contrast T1-weighted [C], SWI [D], DWI [E] and ADC map [F] show high signal lesion T2-weighted [A] and FLAIR [B] images, with no enhancement after gadolinium administration [C], low signal on SWI [D] and restricted diffusion (high signal on DWI [E] and low signal on ADC map). MRI is the modality of choice for assessing suspected diffuse axonal injury even in patients with entirely normal CT of the brain. Note also the bilateral subgaleal haematomas.

TBI is a heterogeneous group of injuries with complex relationships with the mechanism of injury and timing of the forces impacting the skull and body. There are two common assumptions regarding the etiology of mTBI. The first is that the frontal and anterior cortices are vulnerable to neural contusion (Figure 1). The second is that linear and rotational forces act on axon bundles leading to axonal injury^26-29^ (Figures 2 and 3). After the initial injury, secondary mechanisms elicit biochemical, metabolic and cellular changes over the course of minutes, days and months.^30,31^

Traumatic brain injury is the most well-established environmental risk factor for dementia. Currently available data suggest: (I) sufficient evidence of an association between moderate/severe TBI and dementia, with an increased risk of dementia of between 2-and 4-fold for these patients; (II) limited evidence of an association between mild TBI with loss of consciousness and dementia; (III) and inadequate/insufficient evidence to determine whether an association exists between mild TBI without loss of consciousness and dementia.^1,32^ There is no evidence that a single mild TBI episode increases dementia risk. However, as stated before, it is accepted that multiple repetitive mTBIs, as observed in professional boxers, retired professional football, hockey and soccer players and other contact sports players, may have serious long-term consequences.^33^ The risk of dementia in boxers seems most closely tied to the number of rounds boxed, not to the number of times a boxer was knocked out, suggesting that even repeated mild TBIs that do not cause unconsciousness may increase dementia risk.^9^ Additionally, it has been proven that a number of sub-concussive events may be as damaging as a frank concussion.^34^

## PATHOPHYSIOLOGY

TBI initiates an inflammatory cascade that results in the release of amino acids, such as glutamate and aspartate, and free radicals that lead to tissue damage.^35^ Other potential culprits include nitrous oxide, endogenous opioid peptides such as naloxone, catecholamines, acetylcholine, thyrotropin-releasing hormone (TRH), lactate, and adenosine.^36,37^ Cytokines such as tumor necrosis factor (TNF) and interleukins 1,6, and 8, have also been found to increase following TBI.^38^ PET, functional MRI, MR spectroscopy, and SPECT, may play a crucial role in identifying the concentrations and locations of some of these molecules in animal and human brains following injury.^8^

The diffuse axonal injury (DAI) which can occur after severe, moderate and mTBI is an important neuropathological consequence of brain trauma. DAI involves a number of abnormalities ranging from direct damage to the axonal cytoskeleton to secondary damage from disruption of axoplasmic membrane transport, proteolysis, and swelling.^39^ For instance, ionic imbalances, through an efflux of potassium and influx of sodium, lead to calcium influx into cells resulting in mitochondrial damage and impaired oxidative metabolism with lactate production.^16,40,41^

Several investigators have studied the relationship between inheritance of the apolipoprotein ε4 (APOE ε4) allele and dementia (particularly Alzheimer disease) after TBI, with conflicting results. In a population-based study in Northern Manhattan, New York^42^ a history of TBI and inheritance of an APOE ε4 allele were associated with a 10-fold increased risk of dementia while APOE ε4 in the absence of TBI resulted in only a 2-fold increased risk of dementia. The study found no increased risk of dementia due to TBI in the absence of APOE ε4. Similarly, in a prospective study of World War II veterans,^43^ there was a non-significant trend toward higher dementia risk in APOE ε4 carriers. Conversely, in the MIRAGE study,^44^ the influence of head injury on the risk of dementia was greater among persons lacking APOE ε4 compared with those having one or two APOE ε4 alleles.^1^

## PATHOLOGY

In rodent models, TBI resulted in neurodegeneration and progressive brain atrophy that continued for at least 1 year after injury.^1,45^ Several proteins associated with neurodegenerative disease in humans have also been demonstrated to accumulate following experimental TBI in rodents. Amyloid precursor protein is upregulated immediately after TBI and β-amyloid peptide accumulates over weeks and months after trauma.^46-48^ β-Secretase, presenilin 1, and caspase 3 also accumulate for up to 6 months after injury.^47^ In triple transgenic mice expressing pathogenic mutations in amyloid precursor protein, pre-senilin 1 and tau, TBI resulted in accumulations of intra-axonal β-amyloid peptide and hyperphosphorylated tau which persisted for up to 1 week after injury.^49^ These findings have led to the hypothesis that β-amyloid peptide and tau accumulation are important mechanisms in the long-term neurodegenerative effects of TBI. This hypothesis has important therapeutic implications as recent developments in Alzheimer disease (AD) therapeutics, such as anti-β-amyloid antibodies, inhibitors of β- secretase or γ-secretase activity, or other amyloid- or tau-targeted therapies^50,51^ may have potential roles in the management of TBI.^1^

Additional evidence indicating that neurodegeneration after TBI shares some features with AD has been noted on imaging studies. Cerebral atrophy after TBI is not diffuse but rather regionally selective, and the regions that show the most prominent atrophy after TBI, such as the hippocampus, amygdala, precuneus, and parietal and frontal cortices, closely overlap with regions of predominant β-amyloid deposition, decreased glucose uptake, and progressive atrophy in AD.^52^ These findings may be related to common molecular mechanisms shared between AD and TBI-related neurodegeneration.^1,8^ However, AD and CTE are distinct entities. Recent studies in retired professional athletes who had sustained multiple concussions and developed dementia, reported prominent tau-immunoreactive NFTs and astrocytic tangles, but β-amyloid pathology was noted in less than half of the cases.^1,11^ There is also a characteristic distribution pattern of NFTs unique to CTE. The NFTs tend to concentrate in the medial temporal cortex, hippocampus and parahippocampal gyrus, thalamus, mammillary bodies, amygdala, hypothalamus, and substantia nigra. The NFTs also preferentially accumulate in the depths of sulci and surround small blood vessels and in the ventricles in an irregular, patchy distribution. In the neocortices, NFTs concentrate in superficial layers II and III, differing from AD in which NFTs predominate in the deeper layers V and VI. Also unlike AD, there is a paucity of neuritic plaques in CTE.^1,11^

## CLINICAL FINDINGS

**Figure 4:**
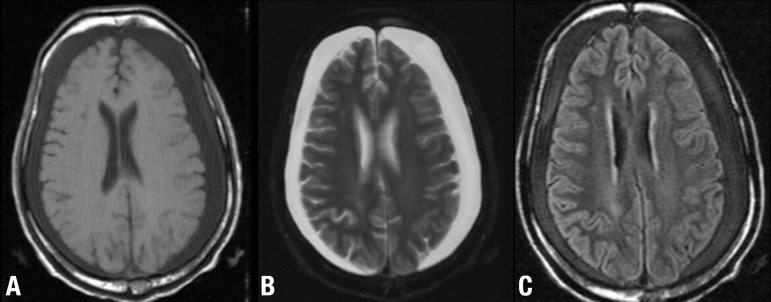
Cognitive decline 8 weeks after TBI. Axial T1-weighted [A], T2-weighted [B] and FLAIR [C] images show bilateral chronic subdural hematomas with no midline shift.

The severity of symptoms depends on whether the injury is mild, moderate or severe. Symptoms often appear at the time of the injury or soon after, but sometimes may not develop for days or weeks. In all levels of TBI, cognitive changes are among the most common, long-lasting and disabling (Figure 4). The ability to learn and remember new information is often affected. Other commonly affected thinking skills include the capacity to pay attention, organize thoughts, plan effective strategies for completing tasks and activities, and make sound judgments.^9^

There is a misconception that loss of consciousness must occur to be characterized as mTBI. As a result, many people with mTBI do not seek help and many health care professionals do not recognize that mTBI has occurred.^16^ Indeed, the term "mild TBI" is actually a misnomer since serious short and long term effects are associated. Individuals with an initial GCS score of 13-15 are acutely at risk for intracranial bleeding and diffuse axonal injury^53^ (Figure 2). Additionally, after a concussion, a minority of patients have persistent disabling symptoms known as post-concussion syndrome (PCS). The rates of PCS vary but most studies report that about 15% to 30% of individuals with a history of a single concussion develop persistent symptoms associated with the injury. The nature of symptoms in PCS may change over time and acute symptoms are most commonly of a physical nature while persistent symptoms tend to be predominantly psychological. Headache and dizziness usually occur immediately after the injury but can also be long lasting. PCS is a clinical diagnosis that is difficult to establish since most symptoms are similar to those experienced by individuals diagnosed with depression, chronic pain, anxiety, or post-traumatic stress disorder.^1,54^ Some experts believe post-concussion symptoms are caused by structural damage to the brain or disruption of neurotransmitter systems resulting from the impact that caused the concussion. Others believe that post-concussion symptoms are related to psychological factors.

PCS should not be confused with CTE. Clinical symptoms of CTE are only beginning to be understood. They are thought to include changes in mood (i.e. depression, suicidality, apathy, anxiety), cognition (i.e. memory loss, executive dysfunction), behavior (i.e. short fuse, aggression), and in some patients, motor disturbances (i.e. difficulty with balance and gait). Differentiating between prolonged PCS and CTE symptoms can be difficult.

## DIAGNOSIS

The diagnosis of TBI in the acute setting is based on neurological examination and neuroimaging tools such as CT and MR imaging. While CT may be critically important in the first 24 hours to assess the immediate need for neurosurgical intervention, MR imaging is more sensitive for detecting small and subtle abnormalities not detected by CT.^19^

In the chronic management of head trauma, imaging has several potential roles such as identifying postoperative neurophysiological sequelae, evaluating the underlying functional abnormalities associated with late complications of head trauma, predicting long-term prognosis, guiding rehabilitation, and developing new therapies to prevent secondary injury.^8,19^

Mild TBI is difficult to diagnose because the brain often appears normal on conventional CT and MR imaging. This lack of imaging evidence of brain injury in mTBI has led clinicians to typically diagnose it on the basis of clinical and cognitive symptoms, which are generally self-reported and are non-specific.^53^ The symptoms may be the result of subtle neurological alterations that are beneath the threshold of what can be detected using conventional neuroimaging techniques.^1,19^ Unlike PCS in which the diagnosis is clinical, CTE diagnosis can only be made at brain autopsy.

Research studies are currently examining whether neuroimaging can detect subtle changes in axonal integrity and structural lesions that can occur in mTBI. Recently, further progress in *in-vivo* diagnostic techniques has been made, particularly with MR imaging advanced techniques. However, more research needs to be done before any such techniques can be validated.^1,21,54,55^

## IMAGING FINDINGS

**MR imaging.** MR imaging of morphometric abnormalities in patients with mTBI has been extensively studied. Generalized brain atrophy is commonly reported and seems to be a common finding in more severely affected patients.^56-64^ Reductions in volume in specific brain regions have been observed including in the hippocampus^65-68^ amygdalas,^63^ fornices,^58,68^ thalamus,^63,67^ and regions of the cingulate gyrus,^59,60,64,67^ as well as enlargements of the lateral ventricles, temporal horns and/or ventricular-to-brain ratio.^58,65,66,69^ Reduced volume in subcortical gray matter regions^58^ and an overall reduction in white matter have also been reported.^57,60,70^ Ding et al.^57^ noted that the changes in white and gray matter over time correlated with acute diffuse axonal injuries and that the latter predicted post-injury cerebral atrophy.^19^

**Figure 5:**
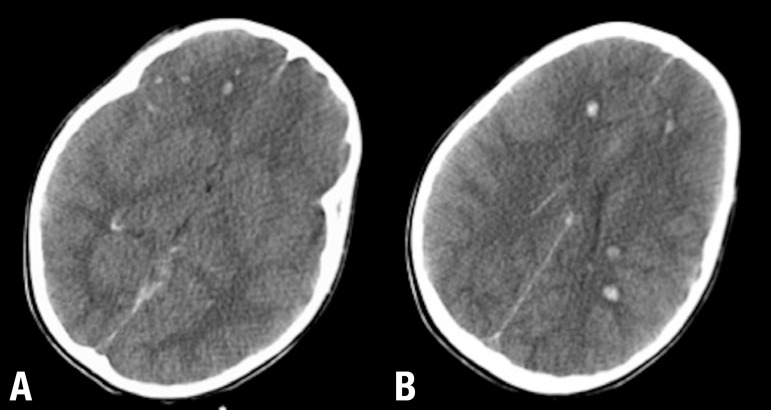
DAI in a patient with severe TBI. Axial non-contrast CT [A and B] show multiple hyperdense lesions varying in size involving primarily the gray-white matter junction. Note that the appearance of DAI on CT depends on whether or not the lesions are hemorrhagic. Subtle or nonhemorrhagic DAI may be not detected on CT and such patients usually have relatively normal CT scans with significant unexplained neurological deficit.

DAI is an important consequence of TBI. DAI is usually diffuse, bilateral and frequently involves the lobar white matter at the gray-white matter interface and may be reversible (Figures 2 and 3). Although DAI is rarely fatal, it can result in significant neurological impairment. The number of lesions correlates with poorer outcomes and lesions in the supratentorial white matter, corpus callosum and corona radiata correlate with a greater likelihood that the patient will remain in a persistent vegetative state. Whereas hemorrhagic axonal injury can be seen on CT as multiple foci of high attenuation, non-hemorrhagic injury can be missed. In fact, CT is abnormal in less than half of all patients with DAI^8^ (Figure 5).

**Figure 6:**
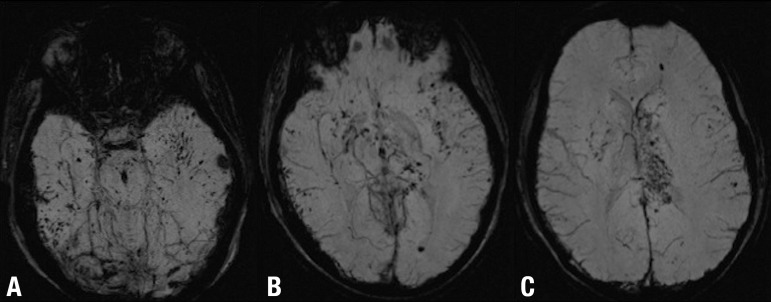
DAI after severe TBI (motor vehicle accident) well depicted on SWI. Axial SWI [A, B and C} shows diffuse and bilateral lesions involving the basal ganglia, lobar white matter, gray-white matter junction, corpus callosum and the brainstem. SWI (or gradient echo sequences) are exquisitely sensitive to blood products and the best MR sequences for DAI detection. Note that when the DAI lesions are entirely nonhemorrhagic they will not be detected on these sequences, but the will be visible as areas of high FLAIR signal.

Susceptibility weighted imaging (SWI) has a six-fold greater ability to detect hemorrhagic DAI than other MR imaging techniques.^71,72^ This technique is thus particularly appropriate for discerning micro-hemorrhages in TBI as it is sensitive to bleeding where small and subtle lesions are not discernible using other MR imaging techniques. This renders SWI particularly useful in the acute and subacute stages following brain trauma^8,19^ (Figure 6).

Diffusion-tensor imaging (DTI) is a relatively new neuroimaging technique that is sensitive to subtle changes in white matter fiber tracts and is capable of revealing microstructural axonal injuries, which may be potentially responsible for persistent post-traumatic symptoms.^1,19^

The concept underlying DTI is that the local profile of the diffusion in different directions provides important indirect information about the microstructure of the underlying tissues. In the white matter, axonal membranes, myelin sheaths, microtubules and neurofilaments restrict the movement of water. This restriction is dependent on the direction of the axons (i.e. diffusion is not equal in all directions). Water diffuses freely in directions parallel to axons but it is restricted in directions perpendicular to axons which results in the magnitude of the diffusion along axons being greater than the two perpendicular directions thus leading to an elongated ellipsoidal shape of the diffusion tensor described as "anisotropic." There are various ways that the shape and size of a diffusion ellipsoid can be quantified but the two most common indices used are Fractional Anisotropy (FA) for shape, and Mean Diffusivity (MD) for size. FA is a scalar measure that ranges from 0 to 1, with 0 indicating complete isotropy meaning that water diffuses equally in all directions and 1 depicting the most extreme anisotropic scenario in which molecules are diffusing along a single axis. Accordingly, in normal white matter FA should be close to 1 and reduced FA is generally thought to reflect loss of white matter integrity. DTI, however, is somewhat non-specific when using these parameters and it is not known whether disruptions in FA and MD are the result of disturbances in axonal membranes, myelin sheath, microtubules, neurofilaments, or other factors.

Different DTI studies in TBI have shown that most of the abnormalities have a systematic spatial distribution corroborating previous suggestions that anterior regions of the brain are more vulnerable to post-traumatic abnormalities.^54,73-75^

Different studies support the hypothesis that acute mTBI is associated with elevated anisotropy values and chronic mTBI with depressed anisotropy.^54,76,77^ However, some studies have reported both trauma-related decreases and increases in FA, particularly in the subacute phase post-injury while many others have reported that FA values increase in both acute and chronic phases of injury.^21^ One possibility for the increase in FA acutely is that MR diffusion-based measurements are not only sensitive to white matter microstructure but additionally are affected in the acute stage by inflammation, secondary injury (ischemia, cerebral hypoxia, and cerebral edema) and/or compensatory mechanisms.^76,77^ Chronically though, residual damage of white matter may lead to decreased anisotropy. Whatever the causative mechanism is, poor neuropsychological performance seems to be associated with high anisotropy scores immediately after injury and with low anisotropy in the chronic phase.^54^

More specific measures to further delineate the biological meaning of alterations in white matter integrity are under investigation including Mode,^78^ which more precisely defines the shape of the diffusion tensor (useful in distinguishing the anatomy of fiber tracts, including distinguishing fiber crossings from pathology); Inter-Voxel Coherence,^79^ which measures how similar anisotropic tensors are in neighboring voxels and is useful for measuring anomalies in macroscopic axonal organization within the tract of interest, and Axial and Radial Diffusivity which are purported to measure axonal and myelin pathology, respectively. Additional approaches include the free-water model^80^ by which it is possible to explicitly estimate the volume of extracellular water, a measure that is sensitive to vasogenic edema and therefore likely specific to neuro-inflammation; Multi-shell diffusion imaging which is a new approach where DWI is acquired at multiple b-values in the same session providing additional microstructural information about the organization of white and gray matter; and kurtosis which is a measure of the deviation from the diffusion tensor model (Gaussian diffusion) and thus complements the other measures derived from the tensor model.^19^ Other methods for resolving crossing fibers include the neurite orientation dispersion and density imaging model,^81^ Q-ball imaging^82^ and constrained spherical deconvolution.^83^ Further studies are required to determine which method is the best.^21^

A normative atlas of DTI-derived measures that depict anatomical variations in healthy controls can also be created so that individual cases may then be compared to discern the pattern of pathology.^19^

In summary, DTI is a promising neuroimaging technique that may help to identify axonal injury after mTBI, however, its role in routine clinical practice has not yet been established

Another promising technique is MR spectroscopy (MRS), which is able to quantify different brain metabolites including N-acetylaspartate (NAA) for neuronal integrity, creatine (Cr) for cellular energy/attenuation, choline (Cho) for membrane turnover, myoinositol an osmotic and gliosis marker, and lactate for anaerobic metabolism. NAA, creatinine, choline, and myoinositol seem to be sensitive to neuronal injury in DAI. In mild TBI, the most common finding is a widespread reduction in gray matter and white matter NAA^21,56,84-87^ and several investigators have found that lower NAA to creatine ratios correlate with poorer clinical outcomes.^8,88^ Studies of MR spectroscopy in chronic mild TBI have found reductions in NAA in the splenium of the corpus callosum,^89^ centrum semiovale,^90^ and frontal white matter.^91^ Chemical shift imaging^92,93^ and whole-brain NAA studies^92^ have also demonstrated NAA reductions in white matter. However, disparity exists between studies in the quantification of Cho. Some studies have found increased Cho in various regions in the brain parenchyma,^86,92,94^ while others have reported the absence of statistical changes in Cho.^87,93,95^ Changes found in Cho, a marker for cellular proliferation and/or tissue damage may reflect diffuse axonal injury.^90,92^ In the acute phase of head injury, choline-containing metabolites may be released as a result of shear injuries and damage to both cell membranes and myelin.^96^ In chronic brain injury, the mechanism for increased Cho is more likely to be related to diffuse glial proliferation, as corroborated by elevated myoinositol that persists for months after injury.^97^ Recent studies have shown changes in Cr.^95,98^ If Cr is affected by mild TBI, metabolite ratio measurements are not accurate because it is difficult to assess whether these changes are due to the metabolite of interest or to Cr. There is significant heterogeneity in the literature and further research is needed to validate this technique for routine clinical use.^21^

MRS and DTI are complimentary given the biochemical and structural focus of each modality. However, few studies have utilized both together. In severe head injury, the combination of these two modalities provides greater diagnostic accuracy for predicting outcome one year following injury^99^ than either MRS or DTI alone. It is likely that this same combination could also provide greater sensitivity to subtle changes in mTBI.^19^

Different studies have used magnetization transfer MRI (MT) to detect white matter abnormalities in different diseases. Through MT, a magnetization transfer ratio can be derived and used quantitatively to measure the structural integrity of tissues. MT changes have been found to be more sensitive than T2-weighted MR imaging in detecting histologic axonal damage in animal models.^100,101^ Bagley et al.^102^ found associations between MT abnormalities and neurological deficits.^8^

**Figure 7:**
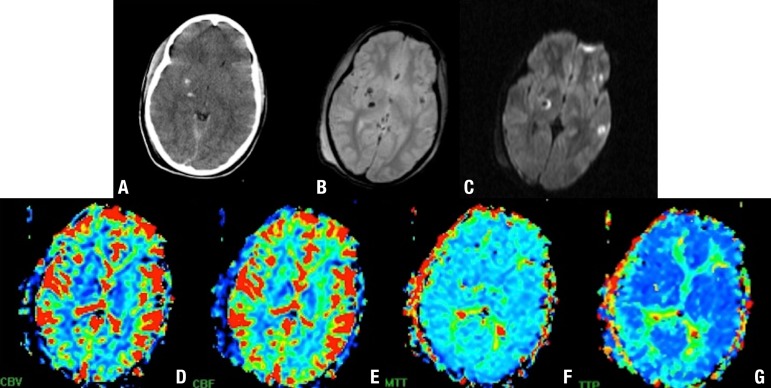
DAI involving the basal ganglia and gray-white matter junction. Axial CT [A], T2 GRE [B] and DWI [C] show hemorrhagic DAI lesions. Perfusion (dynamic susceptibility contrast) MR imaging cerebral blood volume (CBV) [D], cerebral blood flow (CBF) [E], mean transit time (MTT) [F], and time to peak (TTP) [G] maps show low perfusion at the level of the basal ganglia.

Brain perfusion in patients with TBI has been done using different techniques, including stable xenon-enhanced CT, single-photon emission CT (SPECT), PET, perfusion CT, and perfusion-weighted MR imaging (dynamic susceptibility contrast and arterial spin-labeling).^21^ Perfusion-weighted MR imaging is difficult to obtain in the acute setting due to patient contraindications but is an attractive imaging technique for the subacute and chronic phases especially because it can be combined with other MR imaging sequences sensitive to TBI lesions.^21^ In patients with mild TBI and normal conventional brain imaging, perfusion-imaging studies have shown scattered deficits, which correlate significantly with neuropsychological and neurocognitive impairments as well as posttraumatic amnesia (Figure 7). The potential benefits of perfusion imaging in the clinical management of patients with severe TBI have yet to be evaluated. However, patients with altered brain perfusion may be considered for more aggressive and early treatments to prevent intracranial hypertension, whereas patients with preserved brain perfusion may benefit from less invasive treatment. The majority of perfusion studies have involved only limited numbers of patients and further research is required to validate their findings and determine how relevant they are in the management of individual patients with TBI.^21^

Blood oxygen level-dependent (BOLD) fMRI methods for investigating TBI have utilized task-based methods, particularly working memory paradigms. Task- based methods require participation of patients to help identify activation of brain regions thought to drive or be associated with task performances. Task-free resting-state BOLD fMRI techniques have also been used in this setting with the advantage of being able to evaluate distributed whole-brain networks without requiring overt behavioral output from subjects.^21^ Functional MRI studies have found persistent changes in the brain activation patterns of mild TBI patients compared with controls when given various working memory tasks.^8,74^ Additionally, a recent fMRI meta-analysis demonstrated a frontal vulnerability to mTBI as determined by decreased signal in the prefrontal cortex compared to controls. However, there is currently insufficient evidence that fMRI based on BOLD techniques are useful in the routine clinical diagnosis and/or prognosis of TBI.^21^

PET measures cerebral metabolism using different tracers, most commonly 18F-fluorodeoxyglucose (18F-FDG), an analogue of glucose. 18F-FDG measures regional glucose uptake, which should correspond to neuronal viability. PET can also be used in patients with DAI to determine the extent of damage and prognosis since DAI results in diffuse hypometabolism.^103,104^ The major limitation of PET imaging is that it cannot distinguish between functional abnormalities associated or unassociated with structural damage. In general, studies have found that cerebral dysfunction can extend far beyond the boundary of anatomical lesions and may even appear in locations remote from the trauma. Alavi et al.^103^ found that GCS scores of 13 and lower were associated with whole brain hypometabolism on 18F-FDG PET. Additionally, areas of PET cerebral hypometabolism that have no correlates on conventional CT or MRI, are associated with neurological and behavioral dysfunction.^105,106^ Moreover, some of these areas can develop structural abnormalities such as encephalomalacia and atrophy on CT. However, PET imaging can also disclose other potentially confounding neuropsychiatric conditions such as depression or drug induced effects.^8^

SPECT can detect abnormalities in cerebral blood flow (CBF). However, it is not always clear whether the abnormalities observed on SPECT correspond to direct or indirect injuries or possibly abnormalities from prior trauma or other neuropsychiatric conditions. CBF abnormalities are commonly seen in mild TBI patients with chronic symptoms even when no structural damage is apparent.^8,107^

Very few PET and SPECT studies to date have gone beyond CBF and metabolic mapping to incorporate specific ligands for exploring TBI-related changes. Recent PET amyloid imaging suggests that amyloid levels rise in persons with TBI.^36^ A small study with a combined amyloid tau tracer showed higher levels in patients with TBI.^37^ Beta amyloid deposition, while seen in 40%- 45% of chronic traumatic encephalopathy, is not as common as tau accumulation.^21,108^

Only a few studies have used MEG to evaluate the effects of TBI, therefore, more work is necessary to define the utility and capabilities of this imaging technique.^21^

## BIOMARKERS FOR TBI

Over the last decade, there have been numerous studies exploring promising biomarkers of TBI. Despite the large number of publications on this subject there is still no FDA-approved biomarkers for clinical use and further research is needed to introduce them into the clinical setting.^16^

## BIOMARKERS OF ASTROGLIAL INJURY

S100β is considered a marker of astrocyte injury or death. Elevated S100β levels in serum have been associated with increased incidence of post concussive syndrome and cognitive deficits.^6^ However, there is a concern about the specificity of S100β and its utility in the setting of multiple trauma remains controversial since it is also elevated in trauma patients without head injuries.^16,109,110^

Glial fibrillary acidic protein (GFAP) might be a useful marker for various types of brain damage including neurodegenerative disorders, stroke, and severe TBI.^109,111,112^ In a study by Papa et al. in 2012,^113^ GFAP was detectable in serum less than 1 hour after a concussion and was able to distinguish concussion patients from other trauma patients without head injury.^16^

## BIOMARKERS OF NEURONAL INJURY

Neuron-specific enolase (NSE) has been shown be elevated after cell injury. In the setting of DAI in severe TBI, levels of NSE at 72 hours after injury have shown an association with unfavorable outcomes.^114^ One of the limitations of NSE is the occurrence of false-positive results in the setting of hemolysis.^16^

A promising candidate biomarker for TBI currently under investigation is ubiquitin C-terminal hydrolase-L^1^ (UCH- L1). Clinical studies in humans with severe TBI have confirmed that the UCH-L^1^ protein is significantly elevated in human cerebrospinal fluid (CSF),^115^ is detectable very early after injury, and remains significantly elevated for at least 1 week post-injury.^115^ More recently, UCH-L^1^ was detected in the serum of mild and moderate TBI patients within one hour of injury.^113^ Serum levels of UCH-L^1^ discriminated concussion patients from uninjured and non-head-injured trauma control patients that had orthopedic injuries or motor vehicle trauma without head injury.

## BIOMARKERS OF AXONAL INJURY

Alpha-II spectrin (280 kDa) is the major structural component of the cortical membrane cytoskeleton and particularly abundant in axons, and presynaptic terminals of spectrin breakdown products (SBDPs) have been reported in the cerebrospinal fluid (CSF) of adults with severe TBI and shown a significant relationship with severity of injury and clinical outcome.^16,111,116-118^

Tau is an intracellular, microtubule-associated protein that is highly enriched in axons and involved with assembling axonal microtubule bundles and participating in anterograde axoplasmic transport. Because tau is preferentially located in the axon, tau lesions are apparently related to axonal disruption.^16^

Neurofilaments are heteropolymeric components of the neuron cytoskeleton that consist of a 68-kDa light neurofilament subunit (NF-L) backboned with either 160-kDa medium (NF-M) or 200-kDa heavy subunit (NF-H) side arms. After TBI, calcium influx into the cells contributes to a cascade of events that activates calcineurin, a calcium-dependent phosphatase that dephosphorylates neurofilament side arms, presumably contributing to axonal injury.^26^ Phosphorylated NF-H has been found to be elevated in the CSF of adult patients with severe TBI compared with controls.^119^

Although research in the field of TBI biomarkers has increased exponentially over the past 20 years, most studies have focused on severe TBI rather than on mTBI. Because more than 80% of patients have mTBI there is a need to explore biomarkers in this population.^16^

## OUTCOME

Dementia varies widely depending on the cause, age at diagnosis and coexisting health conditions. Post-traumatic dementia is a progressive disorder that worsens over time. Functional outcome depends on how many neurons are preserved after injury. However, the location of damage and the ability of existing neurons to reorganize their connections to recover function are also critical in determining outcomes.^8^

Aside from obvious cases of devastating injuries, a consistent relationship between MRI lesions and clinical or neuropsychological outcomes has not been demonstrated.^8^ In fact, neither CT nor MR imaging findings predict neurocognitive deficits shortly after injury or at 1-year follow-up. Thus, early imaging findings are not predictive of clinical outcome.^120^

SPECT appears to be better than CT or MR imaging for determining long-term prognosis. A negative initial SPECT scan after trauma strongly predicts a favorable clinical outcome^121^ whereas abnormal SPECT is predictive of neuro-psychological deficits.^122^ However, no consistent correlation between SPECT abnormalities and neuropsychological test scores has been established.^123^ Because MR imaging detects lesions missed by SPECT and vice versa, a combination of MR imaging and SPECT may be best for determining prognosis.^8^

Additionally, by combining different biomarkers from blood with MR imaging biomarkers it may be possible to delineate specific types of brain injury involved in mTBI as well as how they change over time and what the evolution of secondary damage or progression of different types of injuries is over time.^19^

## CONCLUSION

There is no known strategy to reduce the possible long-term risks of dementia after a moderate or severe TBI, repeated mild TBIs or even after sub-concussive events, but is important to note that not all individuals who experience head injury develop dementia.

New advanced imaging tools are particularly important for investigating brain pathology in post-traumatic dementia where conventional MR and CT imaging have been found to be insufficient. However, these new techniques are still in the research stage and further investigations are needed before introducing them into clinical routine.
